# Transcription Factor Profiling to Predict Recurrence-Free Survival in Breast Cancer: Development and Validation of a Nomogram to Optimize Clinical Management

**DOI:** 10.3389/fgene.2020.00333

**Published:** 2020-04-24

**Authors:** Hengyu Chen, Xianxiong Ma, Ming Yang, Mengyi Wang, Lei Li, Tao Huang

**Affiliations:** ^1^Department of Pancreatic Surgery, Union Hospital, Tongji Medical College, Huazhong University of Science and Technology, Wuhan, China; ^2^NHC Key Laboratory of Hormones and Development, Tianjin Institute of Endocrinology, Tianjin Medical University Chu Hsien-I Memorial Hospital, Tianjin, China; ^3^Department of Gastrointestinal Surgery, Union Hospital, Tongji Medical College, Huazhong University of Science and Technology, Wuhan, China; ^4^Department of Breast and Thyroid Surgery, Union Hospital, Tongji Medical College, Huazhong University of Science and Technology, Wuhan, China

**Keywords:** signature, TCGA, transcription factor, breast cancer, recurrence-free survival, nomogram

## Abstract

Breast cancer (BC) is the most frequently diagnosed cancer and the leading cause of cancer-related death in young women. Several prognostic and predictive transcription factor (TF) markers have been reported for BC; however, they are inconsistent due to small datasets, the heterogeneity of BC, and variation in data pre-processing approaches. This study aimed to identify an effective predictive TF signature for the prognosis of patients with BC. We analyzed the TF data of 868 patients with BC in The Cancer Genome Atlas (TCGA) database to investigate TF biomarkers relevant to recurrence-free survival (RFS). These patients were separated into training and internal validation datasets, with GSE2034 and GSE42568 used as external validation sets. A nine-TF signature was identified as crucially related to the RFS of patients with BC by univariate Cox proportional hazard analysis, least absolute shrinkage and selection operator (LASSO) Cox regression analysis, and multivariate Cox proportional hazard analysis in the training dataset. Kaplan–Meier analysis revealed that the nine-TF signature could significantly distinguish high- and low-risk patients in both the internal validation dataset and the two external validation sets. Receiver operating characteristic (ROC) analysis further verified that the nine-TF signature showed a good performance for predicting the RFS of patients with BC. In addition, we developed a nomogram based on risk score and lymph node status, with C-index, ROC, and calibration plot analysis, suggesting that it displays good performance and clinical value. In summary, we used integrated bioinformatics approaches to identify an effective predictive nine-TF signature which may be a potential biomarker for BC prognosis.

## Introduction

Breast cancer (BC) is one of the most common malignancies and a leading cause of cancer death among women worldwide (Kwon et al., 2015; Hong et al., 2017; Zhang et al., 2017). Indeed invasive BC is the most frequently diagnosed cancer and leading cause of cancer-related death in young women, with invasive ductal carcinoma being the most common pathological type (Lebeau et al., 2014; Siegel et al., 2014). However, the 5-year survival rate of patients with metastatic BC is around 20% (Chau and Ashcroft, 2004); therefore, the identification of sensitive and specific biomarkers of BC prognosis is essential.

It has become increasingly apparent over the past few decades that tumor biomarker signature is crucial for exploring effective treatments for BC. Clinicopathological parameters, including tumor size, grade, nodal status, and patient age, have been combined to predict prognosis in BC patients; for instance, the Nottingham prognostic index based on tumor size, lymph node stage, and pathological grade serves as the standard in primary BC (Haybittle et al., 1982). The IHC4 index has been developed as a non-commercial algorithm that evaluates four protein markers (*ER*, *PR*, *HER2*, and *Ki67*) to yield a disease recurrence score; however, its clinical application has been impaired due to a lack of validation studies and poor reproducibility (Cuzick et al., 2011; Vieira and Schmitt, 2018). In addition, a recent study that implemented a machine learning approach revealed that microRNAs can serve as biomarkers in BC (Rehman et al., 2019).

Transcription factors (TFs) are DNA-binding proteins that can act as tumor suppressors or oncogenes (Hughes, 2011) by playing crucial roles in the regulation of gene expression and can lead to the avoidance of apoptosis and uncontrolled growth (Bhagwat and Vakoc, 2015). Several prognostic and predictive TF markers have been identified for BC; for instance, the TF *KLF4* has been reported to serve as an independent predictive marker for pathological complete remission in BC following neoadjuvant chemotherapy (Dong et al., 2014). Moreover, a previous study revealed that *TFEts-1* expression can act as an independent prognostic marker for recurrence-free survival (RFS) in BC (Span et al., 2002). However, there are inconsistencies between these sets of markers due to small datasets, the heterogeneity of the disease, and variation in data pre-processing methods. Therefore, a comprehensive and systematic approach for the identification of TFs as effective predictors for BC prognosis is urgently required.

In this study, we analyzed gene expression data and corresponding clinical information for BC from The Cancer Genome Atlas (TCGA) and Gene Expression Omnibus (GEO) databases to identify corresponding TFs and eligible patients and to explore the utility of a TF signature for BC prognosis. By using Kaplan–Meier and receiver operating characteristic (ROC) analysis, we developed and confirmed a novel nine-TF signature for the prognostic assessment of BC with favorable sensitivity and specificity. Finally, we developed and validated a nomogram, which indicated good prognostic value and clinical utility.

## Materials and Methods

### Data Source and Processing

Gene expression data and corresponding clinical follow-up information for patients with BC were downloaded from TCGA using the TCGAbiolinks package (Colaprico et al., 2016) and from the GEO database using the GEOquery package (Davis and Meltzer, 2007). A total of 24,991 genes and 1,097 patients with BC from the TCGA database were included. Cases without prognostic data or non-TF genes were excluded from the subsequent analysis to avoid the analysis of unrelated data. TFs were determined based on the TRRUST database (Han et al., 2018). Raw expression matrix counts were converted to transcripts per million. Genes with no expression in over 20% of the samples were removed. Consequently, 702 TFs and 868 patients with BC were included in the training set (first 70%) and the internal validation set (remaining 30%). The raw GSE2034 and GSE42568 data were preprocessed and normalized using the robust multichip averaging (Irizarry et al., 2003) method in the affy packages (Gautier et al., 2004) of R (v3.6.1). The batch effects between TCGA sequencing data and GEO microarray data were adjusted by “ComBat” function from the “sva” package (Chakraborty et al., 2012). A total of 286 patients in GSE2034 and 104 patients in GSE42568 were included as the external validation sets. The LASSO method was used to identify candidate TFs to predict the RFS of BC patients. The LASSO COX regression model was implemented via a publicly available R package “glmnet”(Friedman et al., 2010) with 1,000 iterations.

### Gene Set Enrichment Analysis and Protein–Protein Interaction Analysis

The TFs identified by univariate Cox regression analysis (*P* < 0.05) in the training dataset were used for Gene Ontology (GO) analysis and Kyoto Encyclopedia of Genes and Genomes (KEGG) pathway enrichment analysis which were conducted using the R clusterProfiler package (Yu et al., 2012). Adjusted *P* values < 0.05 were considered as statistically significant.

The Search Tool for the screening of Interacting Genes website was used to establish the protein–protein interaction (PPI) network with a cutoff criteria of ≥0.4 (Szklarczyk et al., 2019). The genes with the highest degree of correlation with the surrounding genes were selected as key genes according to the PPI network using Cytoscape3.6.0 software (Shannon et al., 2003). Key sub-modules in the PPI network were determined based on the Molecular Complex Detection (MCODE) plugin with recognition criteria of MCODE scores >10 and >10 nodes.

### Statistical Analysis

The relationships between TF expression levels and RFS were investigated using a univariate Cox model to identify TFs associated with RFS. LASSO analysis was then performed to screen candidate key TFs associated with RFS. After further adjustment, multivariate Cox regression was performed on the candidate key TFs to identify TF signatures that evaluate the RFS of patients with BC.

The 868 patients were separated into a training set (*n* = 608) and an internal validation set (*n* = 260). The training cohort was used to identify the prognostic TF signature that was later confirmed in the internal validation set and two external validation sets. A nine-TF prognostic signature was selected by a linear combination of the regression coefficient based on multivariate Cox regression analysis. The following TF risk score formula was used to determine the RFS risk for every patient using the coefficients from the multivariate Cox regression analysis:

Risk⁢Score

=exp⁢(TF1)*β⁢1+exp⁢(TF2)*β⁢2+…⁢…+exp⁢(TFn)*β⁢n,

where “exp” represents TF expression and “β” represents the regression coefficient of the TF. Patients with BC were grouped into high- and low-risk groups using the median risk score as a cutoff. Kaplan–Meier (KM) and log-rank methods were used to compare the survival rates of the groups using the R “survival” package (Moreno-Betancur et al., 2017). The specificity and the sensitivity of the nine-TF prognostic signature was evaluated using time-dependent ROC curves, and the signature was confirmed in the test, training, and validation sets. ROC and KM curves were used to confirm the accuracy and feasibility of the TF model, with a larger AUC indicating a better model for hazard prediction. Stratified analysis was performed using clinical parameters in the entire TCGA set. All ROC and KM curves were plotted using R (version 3.6.1).

### Nomogram Construction

Univariate and multivariate Cox proportional hazard analyses were conducted based on risk score and other clinicopathological factors. Factors with *P* ≤ 0.05 based on multivariate Cox proportional hazard analysis were combined with the nine-TF risk score to build a nomogram in the “rms” R package (FEH, 2015). The prognostic capacity of the nomogram was assessed using the C-index, ROC, and calibration plots, with its outcome indicated by the calibration curve and a 45° line implying a perfect prediction.

## Results

### Clinical Characteristics of the Study Population

**FIGURE 1:**
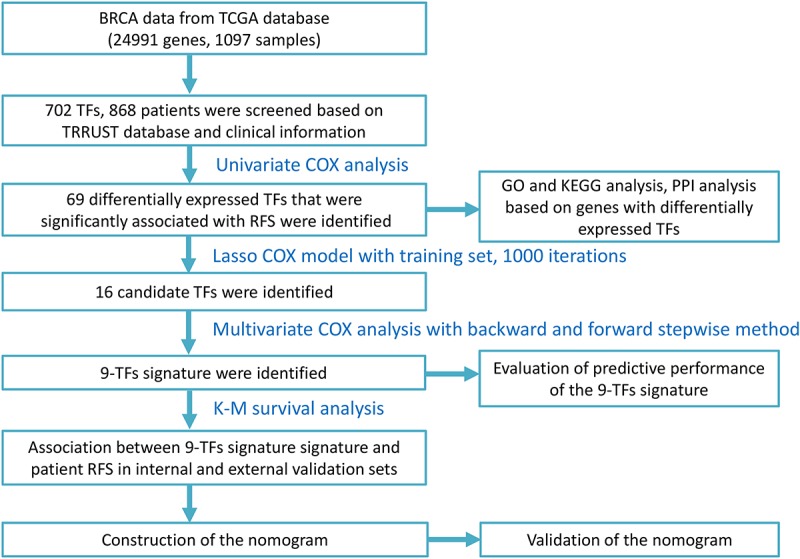
Study design and flow chart.

The study was performed on 868 patients who were clinically and pathologically diagnosed with BC, of which 11 (1.27%) were male and 857 (98.73%) were female. The median age at diagnosis was 57 years (range, 26–90) and the median RFS was 879 days. The 3-year RFS rate of all patients was 41.36%. The pathological stage was defined according to the cancer staging manual of American Joint Committee on Cancer. The stage of the patients with BC ranged from I to V, with 157 (18.1%) patients in stage I, 514 (59.2%) in stage II, 167 (19.2%) in stage III, 14 (1.61%) in stage IV, and 16 (1.8%) in stage X (X – stage not identified). The patients were divided into two groups based on their tumor site: left 462 (53.2%) and right 406 (46.8%). The patients were also separated into three groups according to the margin status of their samples: positive 47 (5.41%), negative 732 (84.33%), and indeterminate 89 (10.3%). The patients’ race list included American Indian or Alaskan Native, Asian, Black or African American, White, and not available, with the majority of patients being White (594, 68.43%). The detailed clinicopathological characteristics of all the patients included in this study are displayed in Table 1. The overall study design and flowchart are shown in Figure 1.

**TABLE 1:** Clinical characteristics of patients with breast cancer who were included in the study.

**Characteristics**	**Total (*n* = 868)**	**Training dataset (*n* = 608)**	**Internal validation dataset (*n* = 260)**	**GSE2034 (*n* = 286)**	**GSE42568 (*n* = 104)**
**Sex**					
Female	857 (98.73)	600 (98.68)	257 (98.85)		
Male	11 (1.27)	8 (1.32)	3 (1.15)		
**Age**					
≤55	395 (45.51)	272 (44.74)	123 (47.31)		46 (44.2)
>55	473 (54.49)	336 (55.26)	137 (52.69)		58 (55.8)
**Tumor**					
T1	242 (27.9)	159 (26.2)	83 (31.9)		
T2	528 (60.8)	379 (62.3)	149 (57.3)		
T3	63 (7.2)	46 (7.6)	17 (6.5)		
T4	33 (3.8)	23 (3.8)	10 (3.8)		
TX	2 (0.23)	1 (0.16)	1 (0.38)		
**Lymph node status**					
N0	398 (45.9)	274 (45.1)	124 (47.7)		
N1	318 (36.6)	225 (37.0)	93 (35.8)		
N2	100 (11.5)	70 (11.5)	30 (11.5)		
N3	40 (4.6)	31 (5.1)	9 (3.5)		
NX	12 (1.38)	8 (1.32)	4 (1.54)		
**Metastasis**					
M0	753 (86.75)	522 (85.86)	231 (88.85)		
M1	16 (1.84)	14 (2.3)	2 (0.77)		
MX	92 (10.6)	66 (10.86)	26 (10)		
**TNM stage**					
Stage I	157 (18.1)	102 (16.8)	55 (21.2)		
Stage II	514 (59.2)	361 (59.4)	153 (58.8)		
Stage III	167 (19.2)	122 (20.1)	45 (17.3)		
Stage IV	14 (1.61)	13 (2.14)	1 (0.38)		
Indeterminate	16 (1.8)	10 (1.6)	6 (2.3)		
**Site**					
Left	462 (53.2)	321 (52.8)	141 (54.2)		
Right	406 (46.8)	287 (47.2)	119 (45.8)		
**Estrogen receptor**					
Positive	600 (69.12)	432 (71.1)	168 (64.6)	209 (73.1)	67 (64.4)
Negative	223 (25.69)	147 (24.2)	76 (29.2)	77 (26.9)	34 (32.7)
Indeterminate	45 (5.2)	29 (4.8)	16 (6.2)		3 (2.9)
**Progesterone receptor**					
Positive	515 (59.3)	369 (60.7)	146 (56.2)		
Negative	305 (35.1)	207 (34.0)	98 (37.7)		
Indeterminate	48 (5.5)	32 (5.3)	16 (6.2)		
**Her2 receptor**					
Positive	139 (16.0)	106 (17.4)	33 (12.7)		
Negative	440 (50.7)	304 (50.0)	136 (52.3)		
Indeterminate	289 (33.3)	198 (32.6)	91 (35)		
**Margin status**					
Positive	47 (5.41)	33 (5.43)	14 (5.38)		
Negative	732 (84.33)	513 (84.38)	219 (84.23)		
Indeterminate	89 (10.3)	62 (10.2)	27 (10.4)		
**Race**					
American Indian or Alaska Native	1 (0.12)	1 (0.16)			
Asian	46 (5.3)	32 (5.26)	14 (5.38)		
Black or African American	155 (17.86)	115 (18.91)	40 (15.38)		
White	594 (68.43)	408 (67.11)	186 (71.54)		
Not available	72 (8.29)	52 (8.55)	20 (7.69)		

### Gene Set Enrichment Analysis and Protein–Protein Interaction Analysis

**FIGURE 2:**
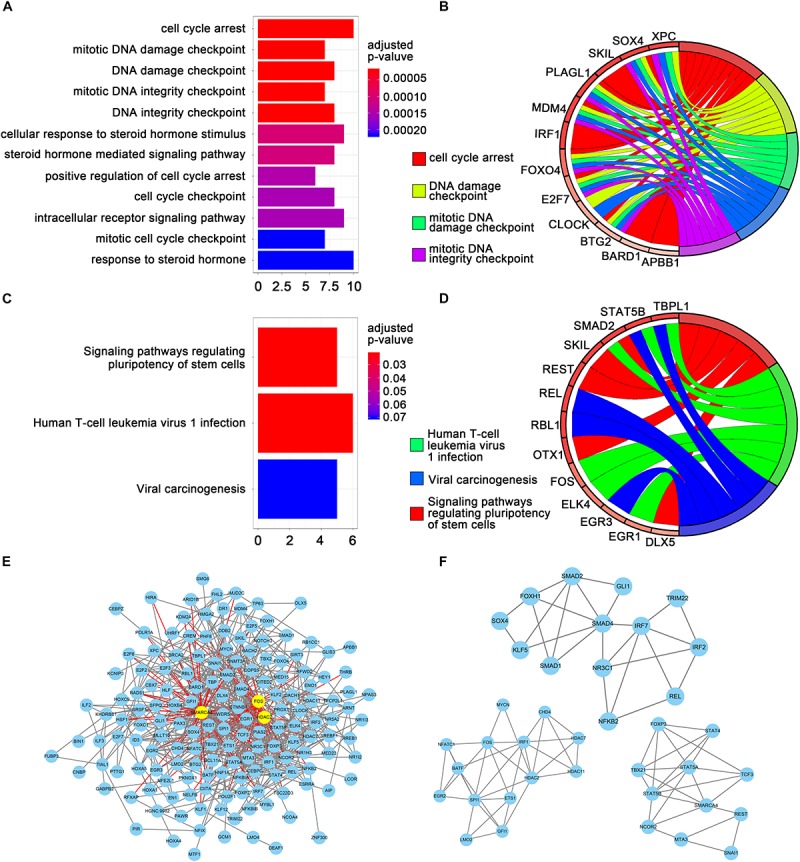
Gene set enrichment analysis and protein-protein interaction (PPI) analysis. **(A)** The top 12 significant GO enrichments for 69 transcription factors. The original adjusted *P* values were transformed by “–log (adjusted *P* value)” to plot the bar chart. **(B)** The top five GO enrichments with gene linkages. **(C)** The top three significantly enriched KEGG pathways. **(D)** The top three KEGG pathways with gene linkages. **(E)** Construction of the protein–protein interaction network of the 69 transcription factors. Yellow nodes represent hub genes. **(F)** The top three sub-module from the PPI network.

Gene ontology and KEGG enrichment analyses were performed using the clusterProfiler package (Yu et al., 2012) to explore the functions and mechanisms of the TFs screened by univariate Cox regression analyses. Figures 2A,B show the top 12 enriched GO terms and top five enriched GO terms with gene linkages. Figures 2C,D show the top three enriched KEGG pathways and the top three enriched KEGG pathways with gene linkages. The top three enriched GO terms were DNA damage checkpoint, mitotic DNA damage checkpoint, and cell cycle arrest. The top three enriched KEGG pathways were signaling pathways regulating the pluripotency of stem cells, human T-cell leukemia virus 1 infection, and viral carcinogenesis (Supplementary Tables S1, S2).

The TFs identified by univariate Cox regression analyses were used to establish the interaction relationships between proteins. Only genes with a combined score >0.4 were used to establish the network. After removing the unmatched genes, 689 pairs of protein relationships were identified. Genes with interactions >10 were considered as hub genes. A total of three hub genes were identified: *HDAC2*, *SMARCA4*, and *FOS* (Figure 2E). All 689 pairs of protein relationships were analyzed using the MCODE plugin, and the top three key sub-modules were used for gene functional annotation. An enrichment analysis found that the genes in those three sub-modules were primarily involved in the regulation of transcription from RNA polymerase II promoter, regulation of DNA-dependent transcription, and regulation of RNA metabolic process (Figure 2F).

### Identification of Transcription Factors Significantly Associated With Recurrence-Free Survival and Establishment of Prognostic Signature

**FIGURE 3:**
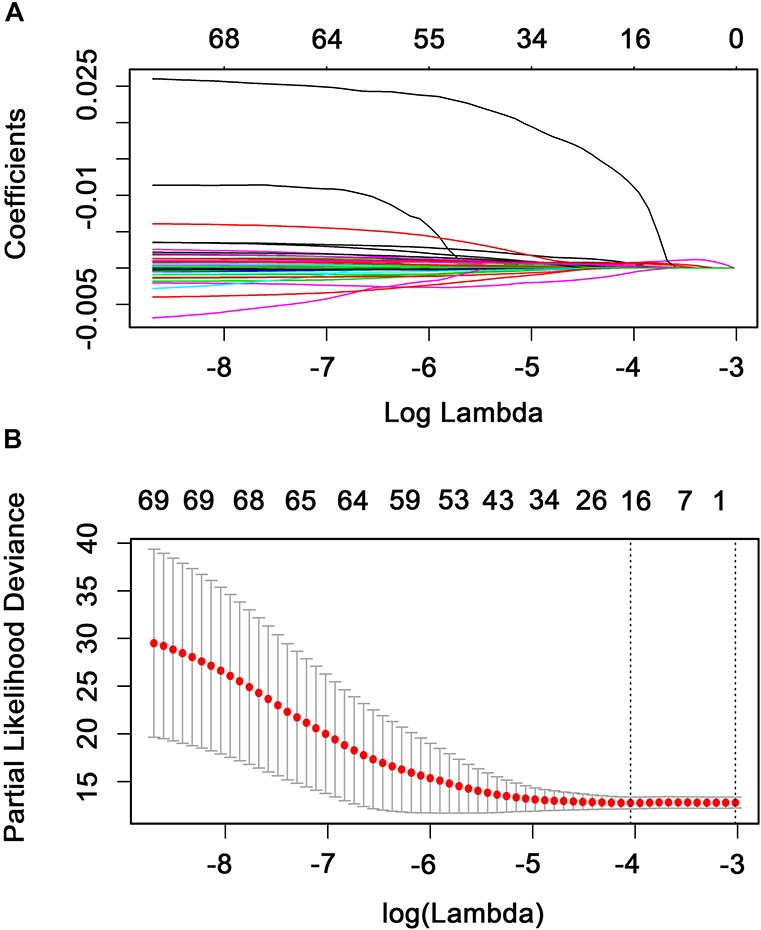
Candidate methylation site selection using the LASSO Cox regression model. **(A)** Tenfold cross-validation for tuning parameter selection in the LASSO model using minimum criteria (1-SE). **(B)** LASSO coefficient profiles of the 69 transcription factors. A coefficient profile plot was produced against the log(lambda) sequence. A vertical line was drawn at the value selected using 10-fold cross-validation where the optimal lambda resulted in 16 non-zero coefficients.

The relationship between the expression level of the 702 TFs and RFS was analyzed using a univariate Cox model. A total of 69 TFs were found to be significantly associated with the RFS of patients with BC (*P* < 0.05; Supplementary Table S3) and were subjected to LASSO analysis to screen key candidate TFs associated with RFS, identifying a total of 16 TFs as candidate prognostic factors for predicting the RFS of patients with BC (Figures 3A,B). Multivariate Cox regression was performed on the 16 candidate TFs to identify TF signatures that evaluate the survival of patients with BC. A nine-TF signature (*FUBP3*, *CLOCK*, *TFCP2L1*, *RFX1*, *PLAGL1*, *TBX2*, *KCNIP3*, *OTX1*, and *BACH2*) was developed and used to predict the RFS of patients with BC using the following risk score formula:

Risk⁢score= 0.00086*F⁢U⁢B⁢P⁢3+ 0.00245*R⁢F⁢X⁢1+ 0.00015

*TFCP2L1+ 0.00146*OTX1- 0.00462*BACH2+ 6e-04*

**FIGURE 4:**
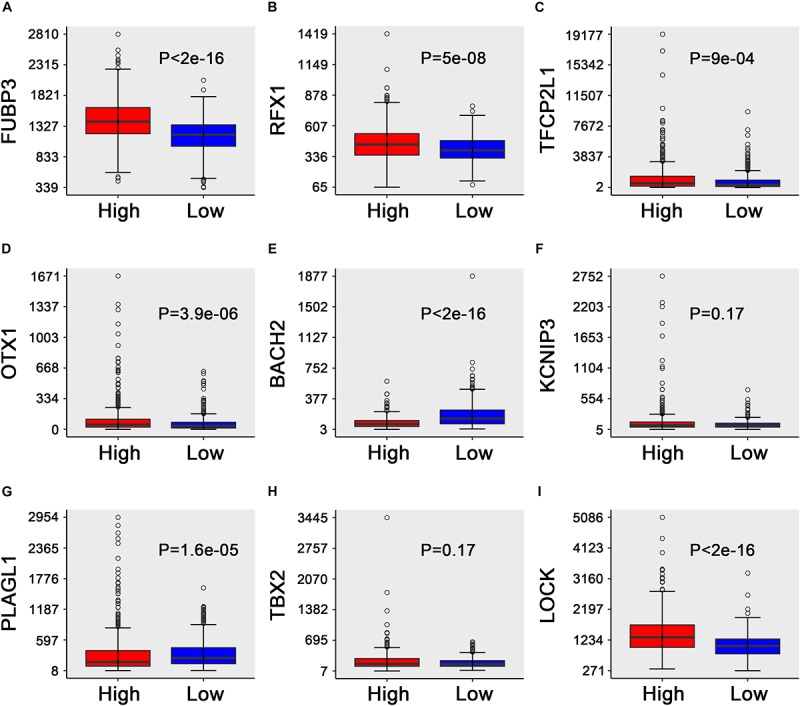
Box plots of the expression of the nine transcription factors against risk group in the TCGA dataset. **(A–I)** “High” and “low” represent the high-risk group and the low-risk group, respectively. The median risk score was taken as a cutoff. The *y*-axis represents the expression level of nine TFs, respectively. The differences between the two groups were estimated using Mann–Whitney *U* tests, with the adjusted *P* values indicated in the graphs.

K⁢C⁢N⁢I⁢P⁢3+ 9⁢e-04*P⁢L⁢A⁢G⁢L⁢1+ 0.001*T⁢B⁢X⁢2+ 0.00074*C⁢L⁢O⁢C⁢K,

where high *FUBP3, RFX1, TFCP2L1, OTX1, KCNIP3, PLAGL1, TBX2*, and *CLOCK* levels and low *BACH2* levels are associated with a higher risk (**Figure 4**). Similar results were obtained in the GSE2034 and GSE42568 sets (**Figure S1-S2**).

### Association Between the Nine-Transcription Factor Signature and Patient Recurrence-Free Survival in the Internal Validation Dataset and Two External Validation Datasets

**FIGURE 5:**
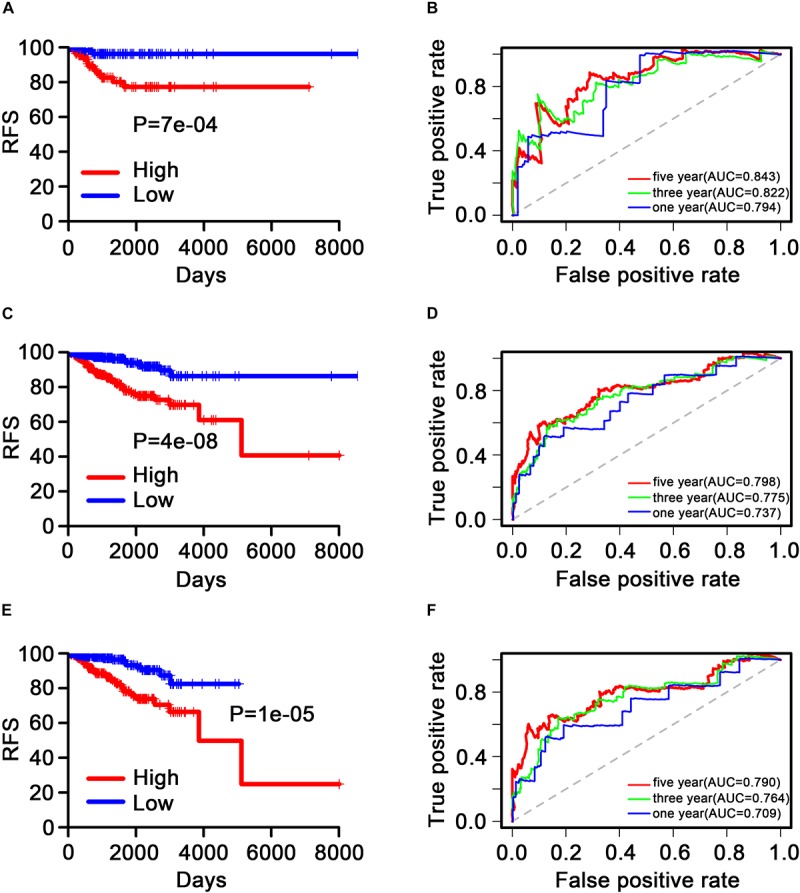
Kaplan–Meier and receiver operating characteristic analysis of patients with breast cancer in the internal validation, GSE2034, and GSE42568 sets. **(A)**, **(C)**, and **(E)** Kaplan–Meier analysis with the two-sided log-rank test was performed to estimate differences in recurrence-free survival (RFS) between the low-risk and the high-risk patients. **(B)**, **(D)**, and **(F)** 1-, 3-, and 5-year receiver operating characteristic curves of the nine transcription factor signatures were used to demonstrate the sensitivity and specificity for predicting the recurrence-free survival of patients with breast cancer. “High” and “low” represent the high-expression group and the low-expression group, respectively. The median risk score was taken as a cutoff. “RFS” represents the relapse-free survival.

The patients were then separated into low-risk (< median value, *n* = 434) and high-risk (> median value, *n* = 434) groups stratified using the nine-TF signature risk score. KM analysis was performed in the internal validation, GSE2034, and GSE42568 sets to assess the RFS of patients in the low-risk group *versus* the high-risk group. The results showed that patients in the high-risk group had a worse RFS based on the KM survival curve in the internal validation dataset (*P* = 7*e*^–4^; Figure 5A), with similar results observed in the GSE2034 (*P* = 4*e*^–8^; Figure 5C), and the GSE42568 (*P* = 1*e*^–5^; Figure 5E) sets.

### Evaluation of the Predictive Performance of the Nine-Transcription Factor Signature Based on Receiver Operating Characteristic Analysis

Time-dependent ROC curves were produced to assess the prognostic ability of the nine-TF signature. The AUC of the nine-TF signature at 1, 3, and 5 years in the internal validation dataset was 0.794, 0.822, and 0.843, respectively (Figure 5B). A high predictive ability was also observed in the GSE2034 (0.737, 0.775, and 0.798; Figure 5D) and the GSE42568 (0.709, 0.764, and 0.790; Figure 5F) sets, indicating that the nine-TF signature may be a good prognostic model for predicting the survival rate of patients with BC.

**FIGURE 6:**
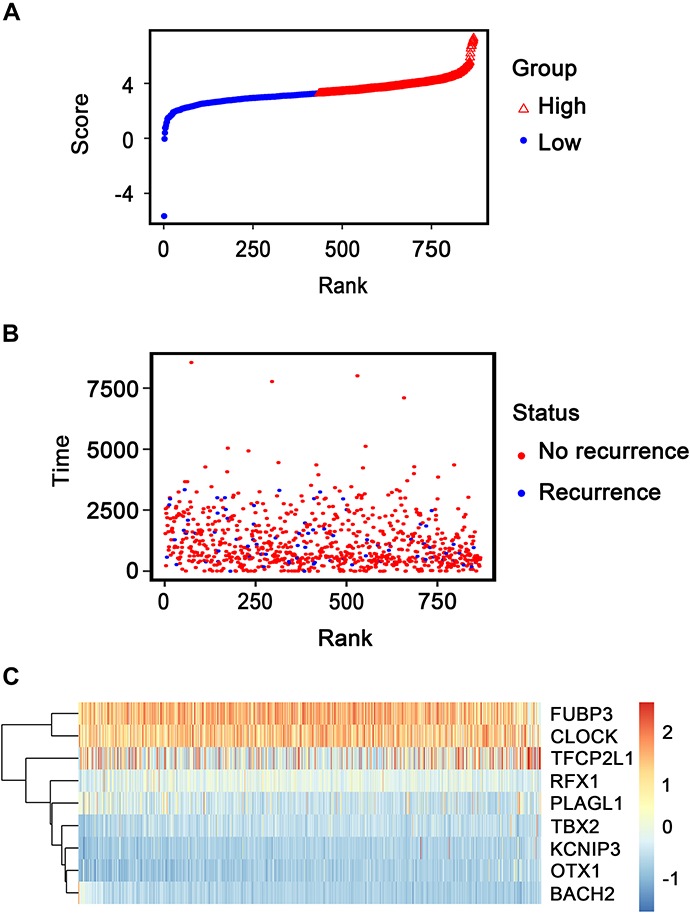
Transcription factor risk score analysis of 868 patients with breast cancer in The Cancer Genome Atlas dataset. **(A)** Transcription factor risk score distribution against the rank of risk score. Median risk score is the cutoff point. **(B)** Recurrence-free survival status of patients with breast cancer. **(C)** Heatmap of the expression profiles of the nine transcription factors in patients with breast cancer.

Patients in the entire TCGA dataset were ranked based on their risk scores (Figure 6A) and a dot plot was produced based on their survival status (Figure 6B), suggesting that the high-risk group had a higher mortality rate than the low-risk group. The heatmap of the nine TFs grouped by risk score (Figure 6C) was consistent with our previous box plot in Figure 4, which has similar results observed in the GSE2034 and the GSE42568 sets (Supplementary Figures S3, S4). The model yielded relatively high AUC values (greater than 0.7) in three independent datasets above, which indicates that this model had a robust predictive performance.

In addition, a subgroup analysis was conducted using several clinicopathological factors, including age, tumor stage, estrogen receptors, progesterone receptors, human epidermal growth factor receptors 2, and metastasis status, indicating that the nine-TF model displayed good performance for predicting BC prognosis in the majority of the sub-groups (Supplementary Figures S5–S10).

### Nomogram Development and Assessment

**FIGURE 7:**
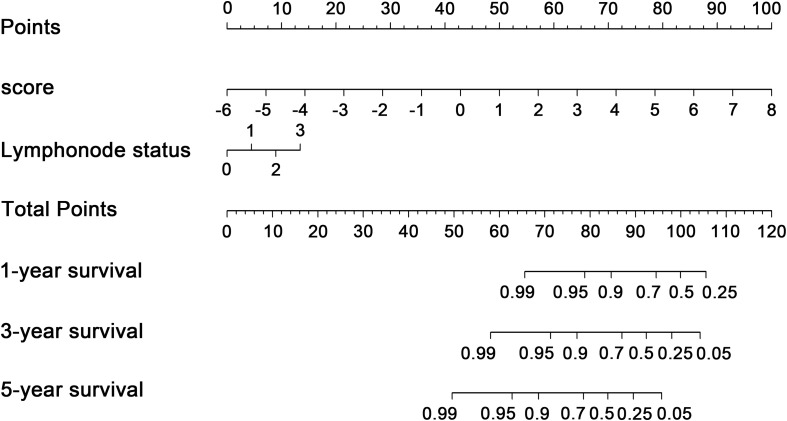
Transcription factor-associated nomogram for the prediction of recurrence-free survival in patients with breast cancer. The nomogram was developed using the entire The Cancer Genome Atlas cohort, with transcription factor risk score and lymph node status.

**FIGURE 8:**
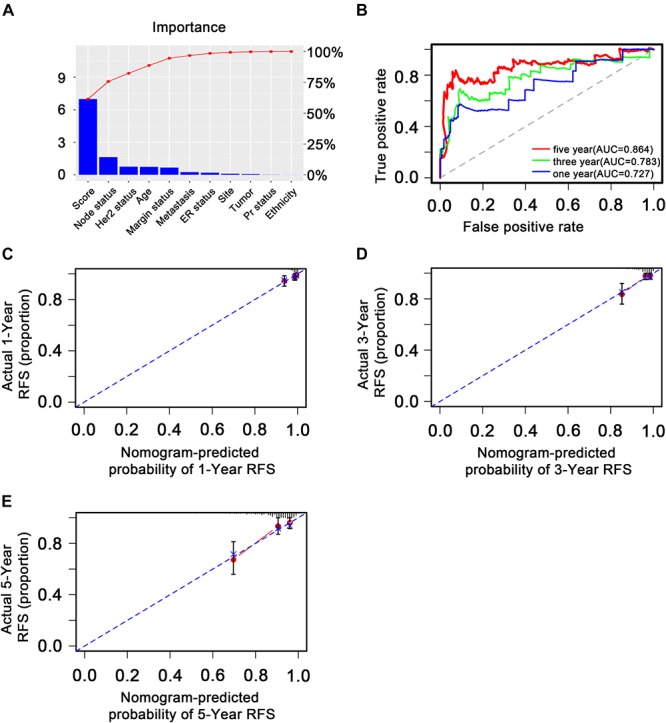
Validation of the transcription factor-associated nomogram in the entire The Cancer Genome Atlas dataset. **(A)** Bar plot of importance of each clinical factor. **(B)** The 1-, 3-, and 5-year receiver operating characteristic curves for the transcription factor-associated nomogram. **(C)**, **(D)**, and **(E)** The 1-, 3-, and 5-year nomogram calibration curves, respectively. The closer the dotted line fit is to the ideal line, the better the predictive accuracy of the nomogram.

To explore whether the nine-TF signature was an independent prognostic predictor for the BC patients’ RFS, we implemented univariate and multivariate Cox models based on the TF-associated risk score and several other clinicopathological factors. The hazard ratios (HRs) showed that the nine-TF signature was significantly related to the BC patients’ RFS (*P* < 0.001, HR 2.51, 95% CI 1.79–3.52; Table 2), suggesting that the nine-TF signature is an independent prognostic indicator. To establish a clinically applicable quantitative method for predicting the BC patients’ RFS, we developed a nomogram which included nine-TF signature and conventional clinicopathological factors (lymph node status) with significant adjusted *P* values in the multivariate Cox model (Figure 7). The importance of each clinical factor is displayed in Figure 8A. According to calibration curve analysis, we found that the 1-, 3-, and 5-year RFS values predicted by the nomogram were closely related to the observed RFS values, which strongly confirmed the reliability of the nomogram (C-index – 0.746; 95% CI, 0.683–0.809; AUC – 1, 3, and 5-year: 0.727, 0.783, and 0.864)(Figures 8B–E).

**TABLE 2:** Univariate Cox regression analysis and multivariate Cox regression analysis outcomes based on methylation risk score and other clinical factors.

	**Univariate Cox regression analysis**				**Multivariate Cox regression analysis**			
**Characteristics**	**HR**	**HR.95L**	**HR.95H**	***p* value**	**HR**	**HR.95L**	**HR.95H**	***p* value**
Score	2.738045421	2.048628528	3.659469066	1.01E-11	2.510661624	1.788829617	3.523768687	1.02E-07
Estrogen receptor status	0.692324993	0.342239438	1.400522099	0.306355543	0.768993041	0.260500851	2.270051305	0.634356191
Progesterone receptor status	0.592458976	0.30230991	1.161085454	0.127286775	0.981504947	0.33480194	2.877378671	0.972862011
Her2 receptor status	1.257944488	0.823912985	1.9206207	0.287844565	1.373910032	0.865382257	2.181265863	0.178008111
Tumor	1.754711187	0.617255141	4.988231197	0.291488237	1.101502581	0.3254944	3.727584669	0.87648392
Lymph node status	2.17751336	1.49200771	3.177975825	5.48E-05	1.651534043	1.069601814	2.550074858	0.023602411
Metastasis status	2.520159824	0.343448624	18.49244717	0.363359385	1.858617241	0.226521566	15.25001838	0.563807742
Margin status	0.7974526	0.396241415	1.604907073	0.525907749	0.634209273	0.307262431	1.309048428	0.218093787
Ethnicity	0.634959689	0.086536008	4.659029397	0.655117076	1.014927373	0.132510023	7.773582306	0.988619118
Age	0.97574987	0.948136617	1.004167323	0.093730595	0.979959839	0.95094581	1.009859108	0.186780098
Anatomic neoplasm subdivision	1.039911088	0.843603069	1.281900351	0.713889548	0.97189382	0.782429155	1.207237219	0.796654191

## Discussion

In this study, we analyzed the gene expression data (24,991 genes) and the clinical data of patients with BC (1,097 patients) from TCGA, using 702 TFs and 868 patients with BC to systematically identify an effective predictive TF signature for the prognosis of patients with BC using bioinformatics methods. A linear combination of nine TFs (*FUBP3*, *CLOCK*, *TFCP2L1*, *RFX1*, *PLAGL1*, *TBX2*, *KCNIP3*, *OTX1*, and *BACH2*) was identified as an independent predictor of the survival of patients with BC. This nine-TF signature was found to have significant prognostic roles in patients with BC, indicating that the nine TFs may have underlying roles in the molecular pathogenesis, clinical progression, and prognosis of BC and may have the potential to improve the clinical prognosis of patients with BC.

Studies have suggested that these nine TFs may play important roles in cancer development. For instance, *SIAH-1* has been reported to positively and indirectly regulate *FBP-3* (*FUBP3*), which primarily supports human hepatocellular carcinoma cell proliferation (Brauckhoff et al., 2011), suggesting a key role in cancer development. The downregulation of *RFX1* has been shown to predict poor prognosis in patients with small hepatocellular carcinoma (Liu et al., 2018), while *TFCP2/TFCP2L1/UBP1* has been found to act as a TF in cancer (Kotarba et al., 2018). In addition, *OTX1* and *OTX2* have been identified as two possible molecular markers for sinonasal carcinomas and olfactory neuroblastomas (Micheloni et al., 2019), whereas *BACH2* is associated with the neuronal differentiation of N1E-115 neuroblastoma cells (Shim et al., 2006). The disruption of repressive p130-DREAM (*KCNIP3*) complexes by human papillomavirus 16 E6/E7 oncoproteins has been found to be essential for cell cycle progression in cervical cancer cells (Nor Rashid et al., 2011). Moreover, *PLAGL1* (*ZAC1*/*LOT1*) expression has been shown to be associated with disease progression and unfavorable prognosis in clear cell renal cell carcinoma (Godlewski et al., 2016). Furthermore, *TBX2* acts as a neuroblastoma core regulatory circuitry component that promotes *MYCN*/*FOXM1-*mediated reactivation of *DREAM* targets (Decaesteker et al., 2018), while *CLOCK* genes are closely associated with cancer development, particularly in endocrine tissues (Angelousi et al., 2019).

A GO and KEGG pathway analysis of the TFs identified by univariate Cox regression analyses identified that the enriched signaling pathways of cell cycle arrest, signaling pathways regulating pluripotency of stem cells, and human T-cell leukemia virus 1 infection were significantly associated with cancer development. The cellular responses to DNA damage are comprehensively known as DNA damage response (DDR) and include DNA repair pathway activation, cell cycle arrest, and cell death induction (Zhou and Elledge, 2000; Bassing and Alt, 2004). The DDR plays a significant role in the field of cancer therapy as both chemotherapy and radiotherapy are based on DNA damage-induced tumor cell death (Jun et al., 2016), which has revealed the key role of cell cycle arrest in cancer therapy. A previous study suggested that BC is significantly associated with signaling pathways that regulate stem cell pluripotency (Wang et al., 2018), indicating that these signaling pathways play a key role in the pathogenesis of BC. Moreover, a study reported that the human T-cell leukemia/lymphotropic virus-1 is the etiological agent of adult T-cell leukemia, an aggressive and fatal leukemia of CD4+ T lymphocytes (Mamane et al., 2002). In combination with our findings, these studies suggest that cell cycle arrest, signaling pathways regulating the pluripotency of stem cells, and human T-cell leukemia virus-1 infection may be useful therapeutic targets for BC; however, additional studies are required to confirm this hypothesis.

We identified three hub genes (*HDAC2*, *SMARCA4*, and *FOS*) by PPI analysis that previous studies have suggested may be crucial in cancer development. For instance, *HDAC2* overexpression has been associated with aggressive clinicopathological features and the DDR pathway in BC (Shan et al., 2017). Moreover, a study found that high *SMARCA4* or *SMARCA2* expression is frequently associated with opposite prognoses in BC (Guerrero-Martinez and Reyes, 2018), while high *FOS* expression has been associated with better BC prognosis (Fisler et al., 2018). Due to the different screening pipelines, there is no significant correlation between the three hub genes and the above nine predictive TFs. Our results also confirmed that these genes do not share the same overlaps. The nine predictive TFs are involved in RFS and might primarily play a vital role in predicting the prognosis of patients with BC, while the three hub genes are likely to play an important role in cancerogenesis or the development of cancer.

Chen et al. used miRNA profiling followed by qRT-PCR confirmation to identify a four-miRNA signature which may act as a potential predictor for the metastasis and the prognosis of patients with BC (Chen et al., 2018); however, this study did not perform GO and KEGG analysis on the targets, unlike ours, and the four-miRNA signature may not have a universal prognostic applicability due to the small datasets used.

Our study has several advantages. For instance, in this study, we performed GO and KEGG analysis to explore the functions and mechanisms of the nine TFs in the progression of BC. Moreover, we used a large gene expression and clinical dataset for BC downloaded from TCGA. We also used the LASSO method to filter variables between the univariate and multivariate Cox analysis, avoiding multicollinearity interference and making our results more reliable. Furthermore, until now, no studies have yet combined a TF signature with clinical indicators to predict RFS for BC; however, we combined TF bioinformatics analysis with clinical indicators to offer a novel method for clinical prediction. In addition, we built a nomogram integrating both the nine-TF signature and the conventional clinicopathological factors to predict 3- and 5-year RFS. We revealed that the nine-TF signature plays significant prognostic roles in clinical patients with BC, making our study highly valuable.

Despite the beneficial outcomes of our study, it had several limitations. Firstly, the samples were randomly divided into training and testing sets for the development and the assessment of the prognostic model; therefore, more independent external validation sets with long-term follow-ups to provide a realistic assessment of the performance of this TF signature would be more reliable. Secondly, the nomogram model was constructed based only on TCGA dataset due to the incomplete clinical information in the GEO dataset. Thirdly, the prognostic value of the nine-TF signature must be further improved and validated in clinical practice.

## Data Availability Statement

All data generated or analyzed during the present study are included in this published article or are available from the corresponding author on reasonable request.

## Author Contributions

HC and XM designed, extracted, analyzed, and interpreted the data from TCGA and GEO databases. MY and MW wrote the manuscript. TH and LL made substantial contributions to the conception of the work and substantively revised it. All the authors have read and approved the final manuscript.

## Conflict of Interest

The authors declare that the research was conducted in the absence of any commercial or financial relationships that could be construed as a potential conflict of interest.
