# Supplementary material for: Transcription Factor Profiling to Predict Recurrence-Free Survival in Breast Cancer: Development and Validation of a Nomogram to Optimize Clinical Management
Source: Front Genet. 2020 Apr 24;11:333. doi: 10.3389/fgene.2020.00333 (PMC7193038; doi:10.3389/fgene.2020.00333)
Supplement: Supplementary file 4 [file Data_Sheet_1.docx]

**Supplementary Figure 1.** Boxplots of nine transcription factor expression values against risk group in the GSE2034 set.

**Supplementary Figure 2.** Boxplots of nine transcription factor expression values against risk group in the GSE42568 set.

**Supplementary Figure 3. Transcription factor risk score analysis of 286 patients with breast cancer in the GSE2034 set.** **(A)** Transcription factor risk score distribution against the rank of risk score. Median risk score is the cut-off point. **(B)** Recurrence free survival of patients with breast cancer. **(C)** Heatmap of nine transcription factor expression profiles of patients with breast cancer.

**Supplementary Figure 4.** **Transcription factor risk score analysis of 104 patients with breast cancer in the GSE42568 set.** **(A)** Transcription factor risk score distribution against the rank of risk score. Median risk score is the cut-off point. **(B)** Recurrence free survival of patients with breast cancer. **(C)** Heatmap of nine transcription factor expression profiles of patients with breast cancer.

**Supplementary Figure 5.** **Kaplan-Meier and receiver operating characteristic analysis of patients with breast cancer in sub-groups according to age.** **(A, B)** Sub-group aged less than 55 years. **(C, D)** Sub-group aged over 55 years.

**Supplementary Figure 6.** Kaplan-Meier and receiver operating characteristic analysis of patients with breast cancer in sub-groups according to stage. **(A, B)** Early stage sub-group. **(C, D)** Advanced stage sub-group.

**Supplementary Figure 7.** Kaplan-Meier and receiver operating characteristic analysis of patients with breast cancer in sub-groups according to estrogen status. **(A, B)** Estrogen negative sub-group. **(C, D)** Estrogen positive sub-group.

**Supplementary Figure 8.** Kaplan-Meier and receiver operating characteristic analysis of patients with breast cancer in sub-groups according to HER2 status. **(A, B)** HER2 negative sub-group. **(C, D)** HER2 positive sub-group.

**Supplementary Figure 9.** Kaplan-Meier and receiver operating characteristic analysis of patients with breast cancer in sub-groups according to progesterone status. **(A, B)** Progesterone negative sub-group. **(C, D)** Progesterone positive sub-group.

**Supplementary Figure 10.** Kaplan-Meier and receiver operating characteristic analysis of patients with breast cancer in sub-groups according to margin status. **(A, B)** Margin negative sub-group. **(C, D)** Margin positive sub-group.
