# Supplementary figures and images for: Transcription Factor Profiling to Predict Recurrence-Free Survival in Breast Cancer: Development and Validation of a Nomogram to Optimize Clinical Management
Source: Front Genet. 2020 Apr 24;11:333. doi: 10.3389/fgene.2020.00333 (PMC7193038; doi:10.3389/fgene.2020.00333)

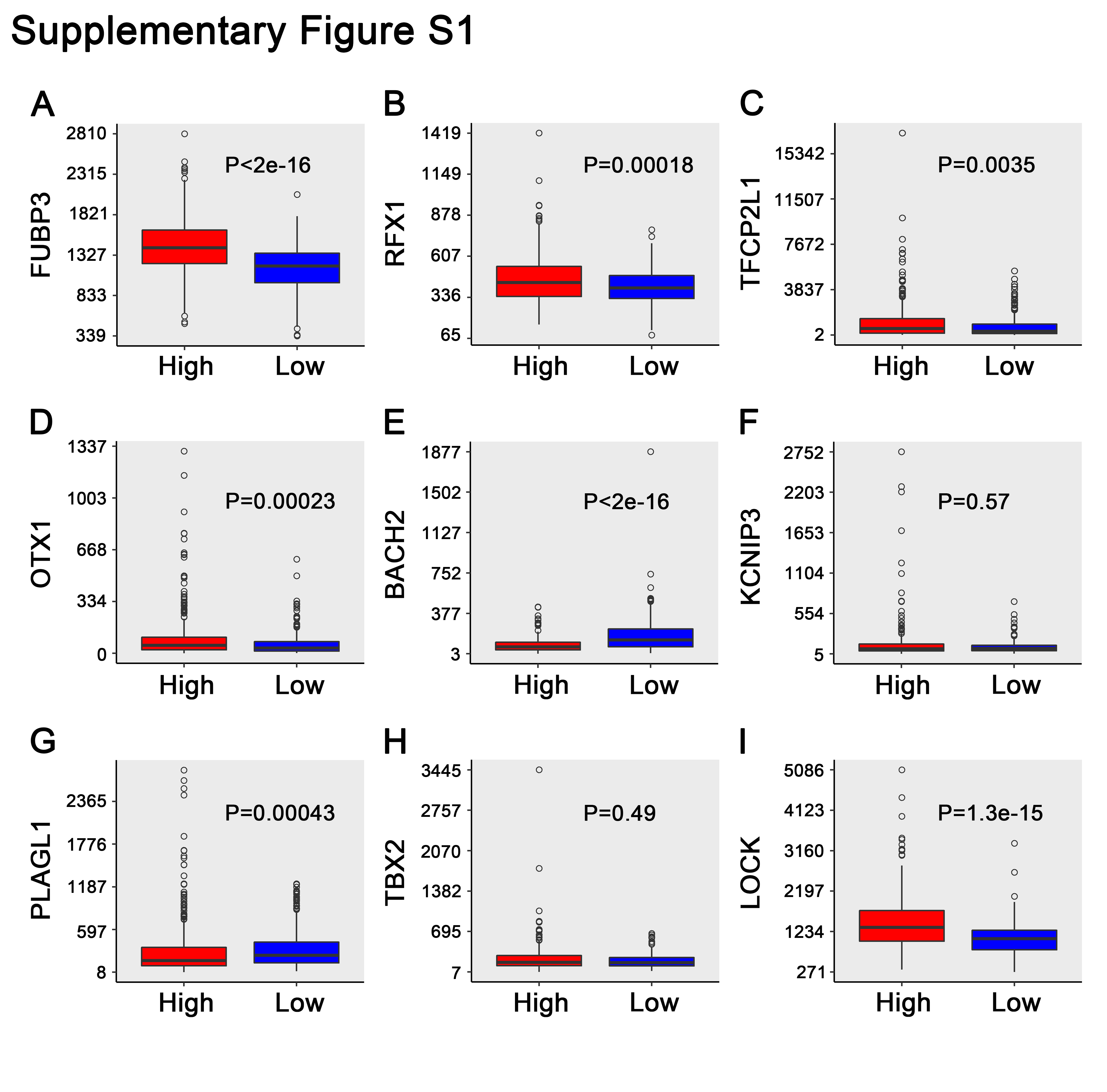

Supplement: Supplementary file 5 [file Image_1.TIF]

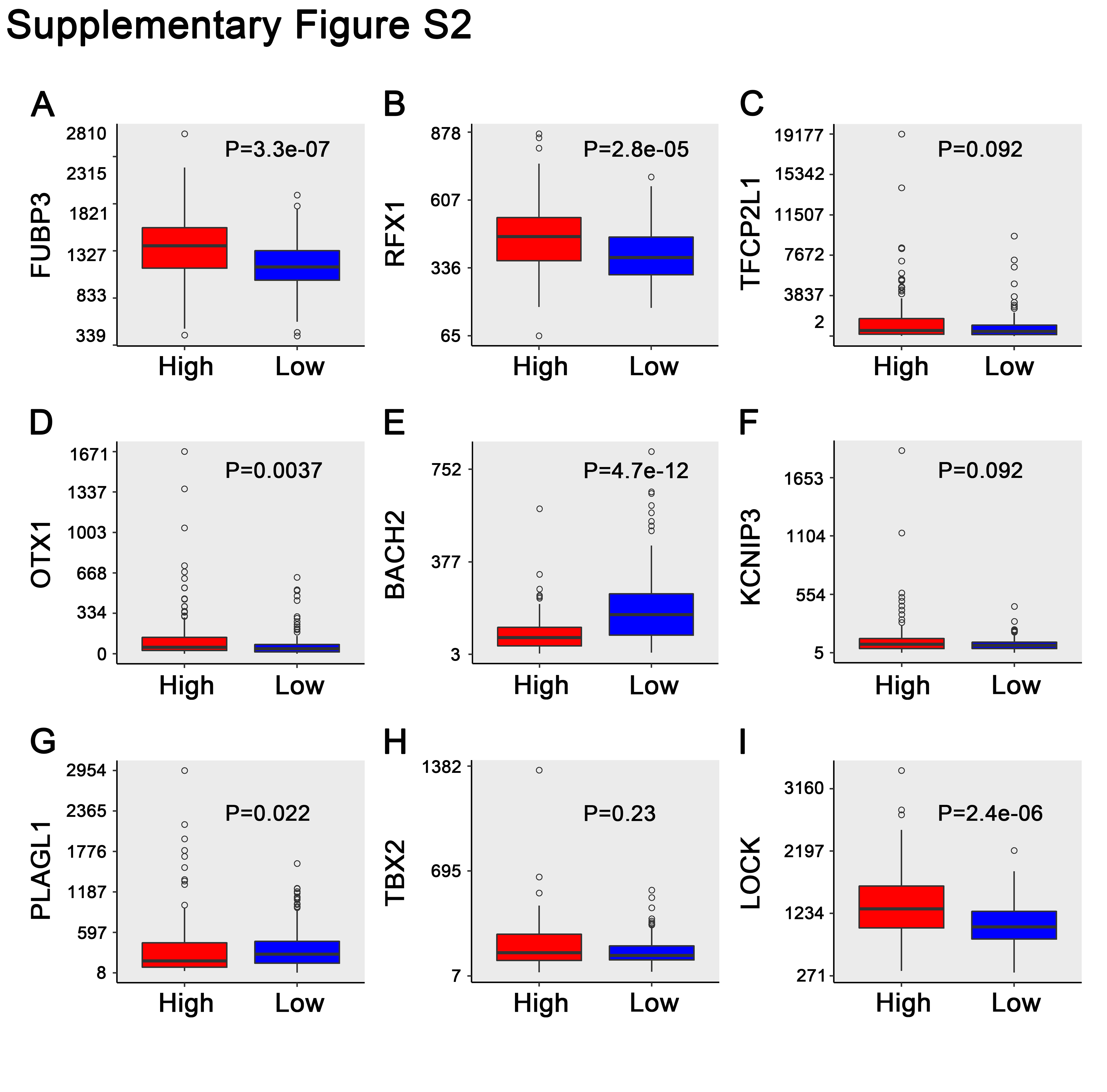

Supplement: Supplementary file 6 [file Image_2.TIF]

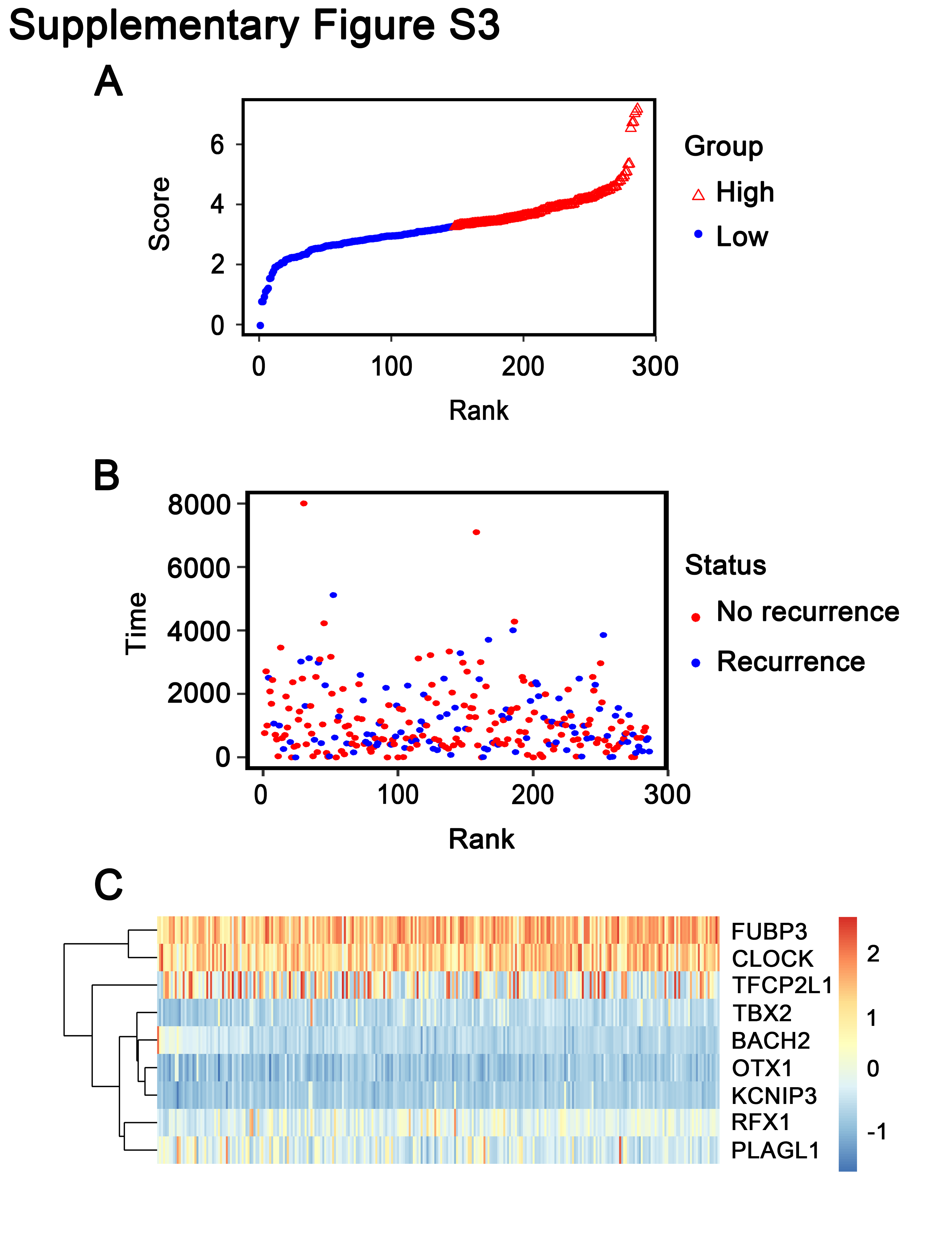

Supplement: Supplementary file 7 [file Image_3.TIF]

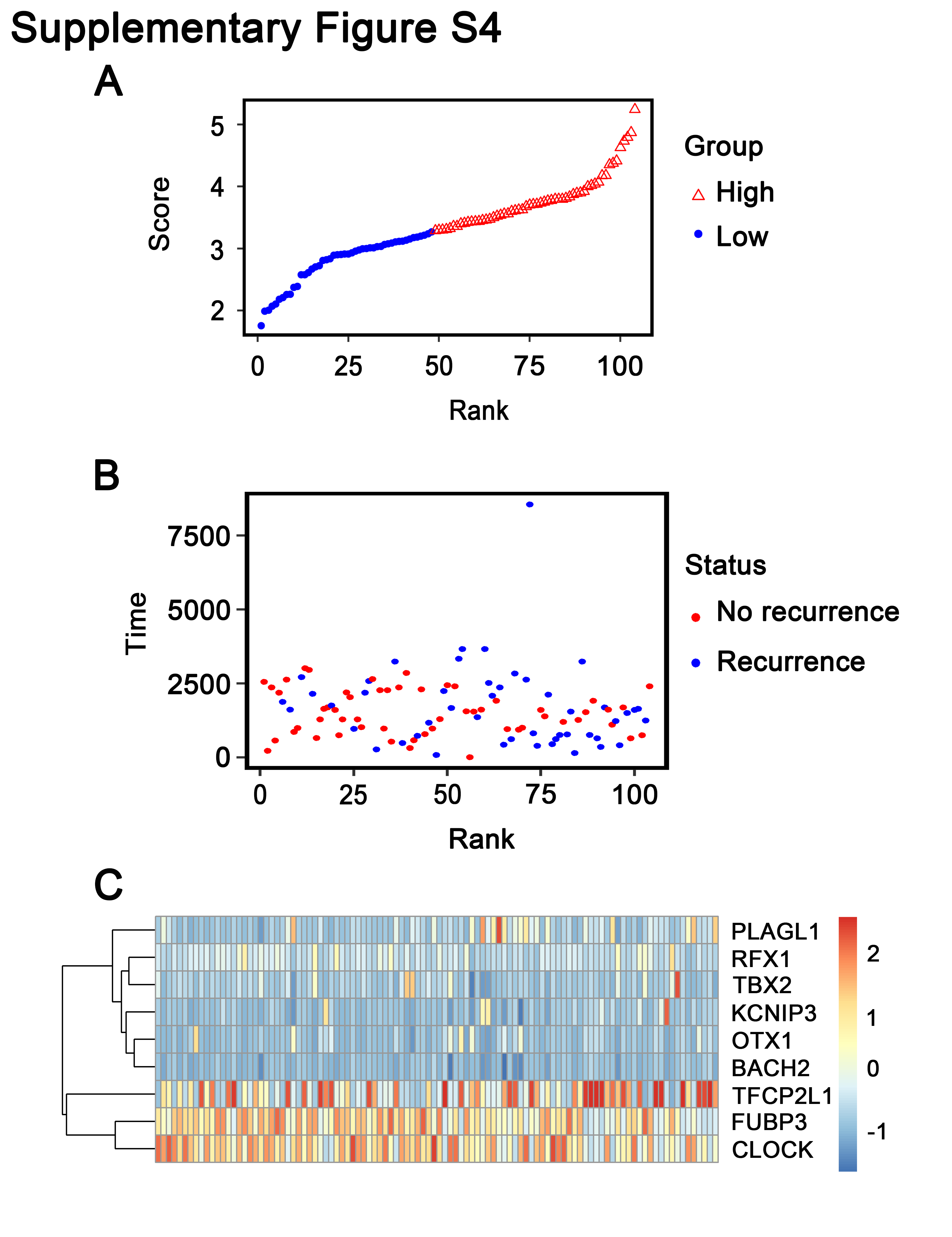

Supplement: Supplementary file 8 [file Image_4.TIF]

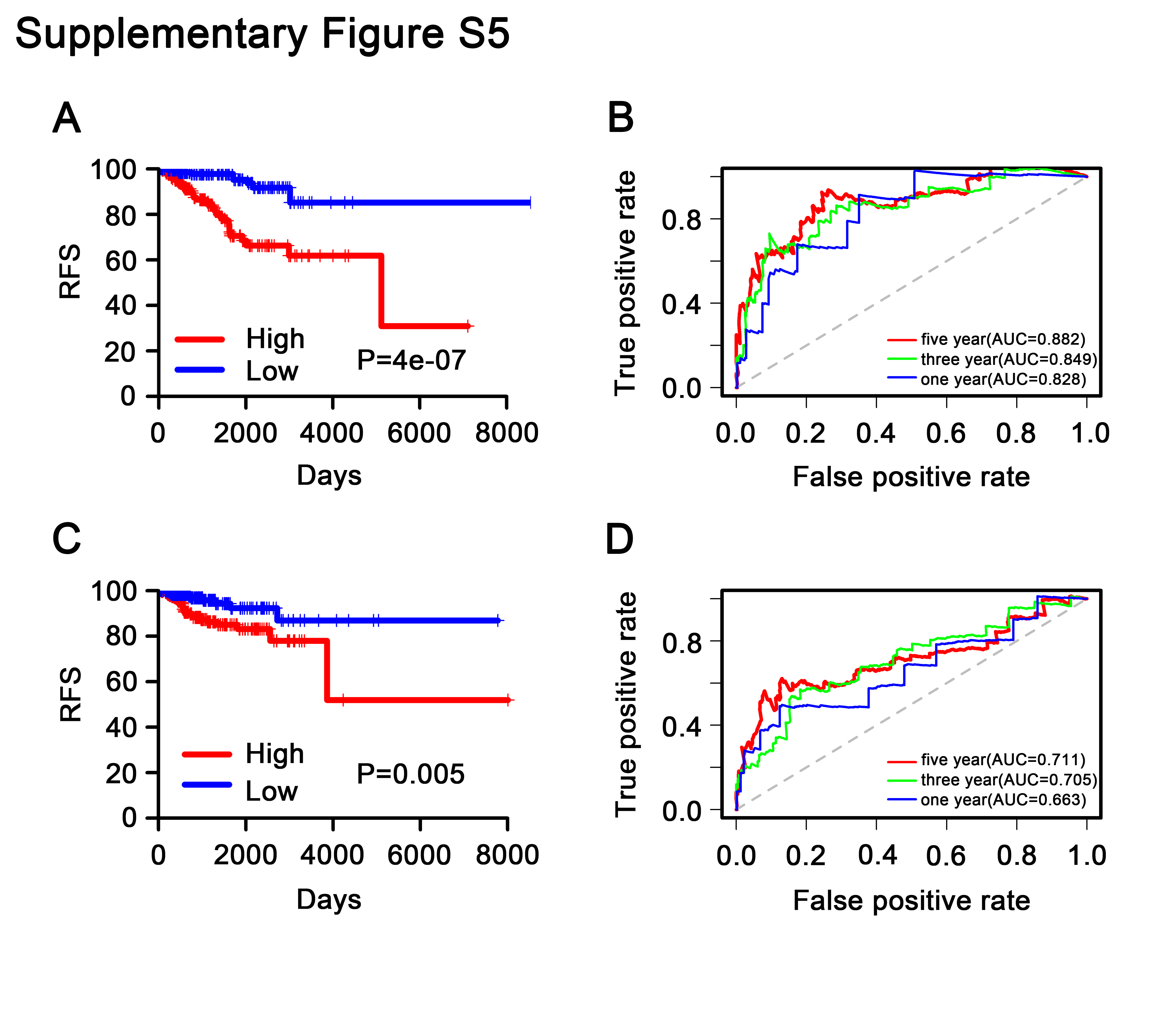

Supplement: Supplementary file 9 [file Image_5.TIF]

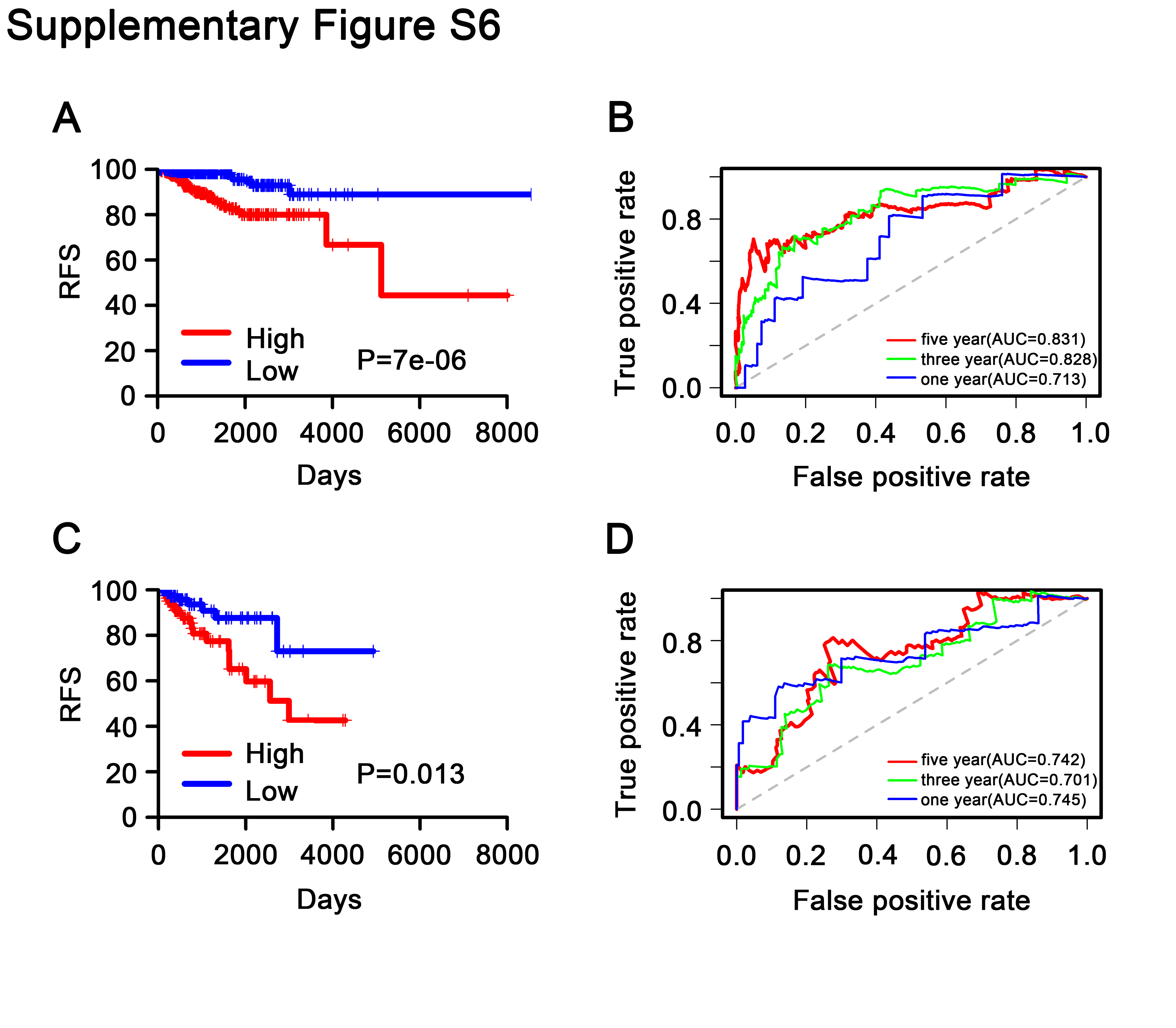

Supplement: Supplementary file 10 [file Image_6.TIF]

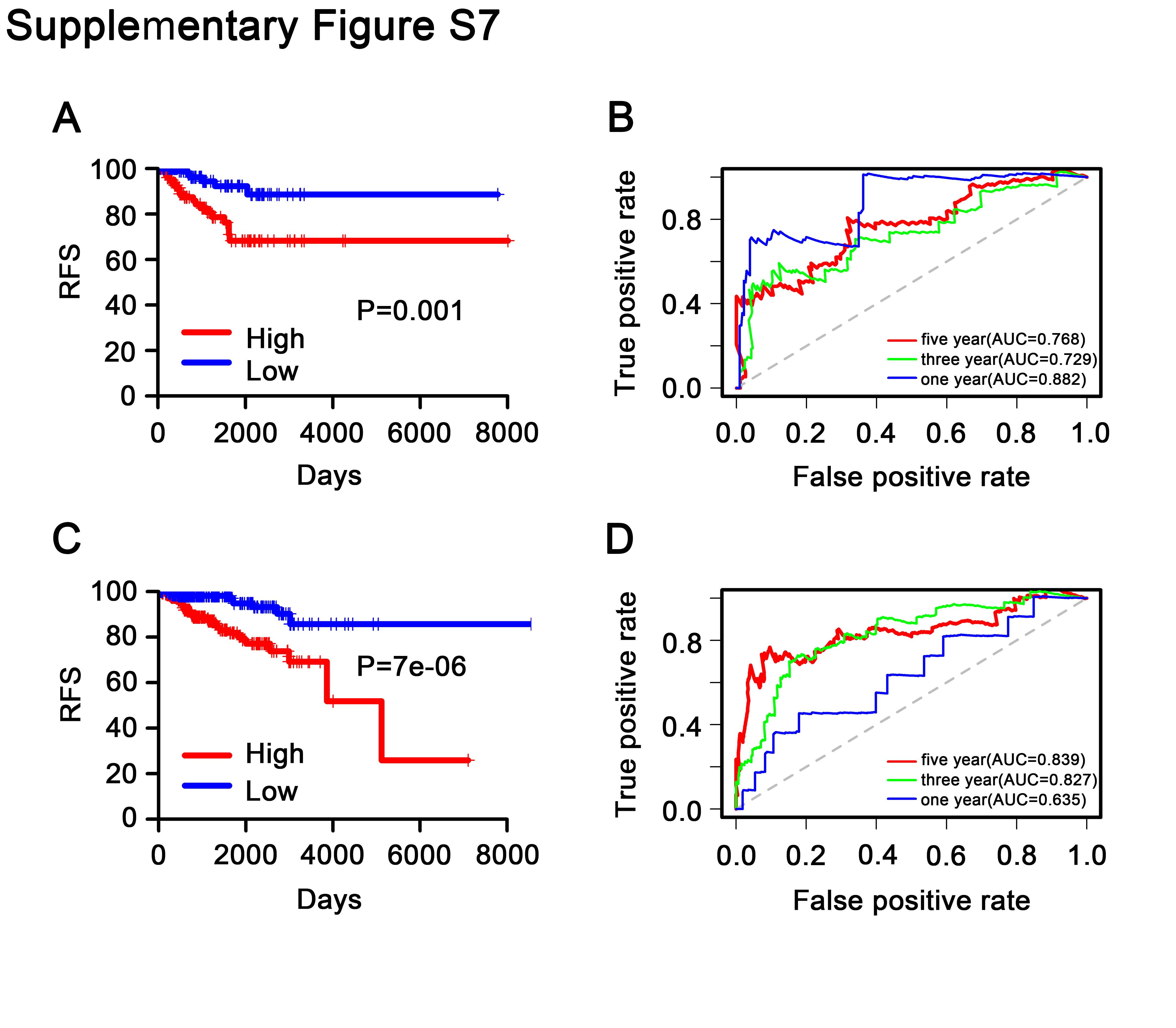

Supplement: Supplementary file 11 [file Image_7.TIF]

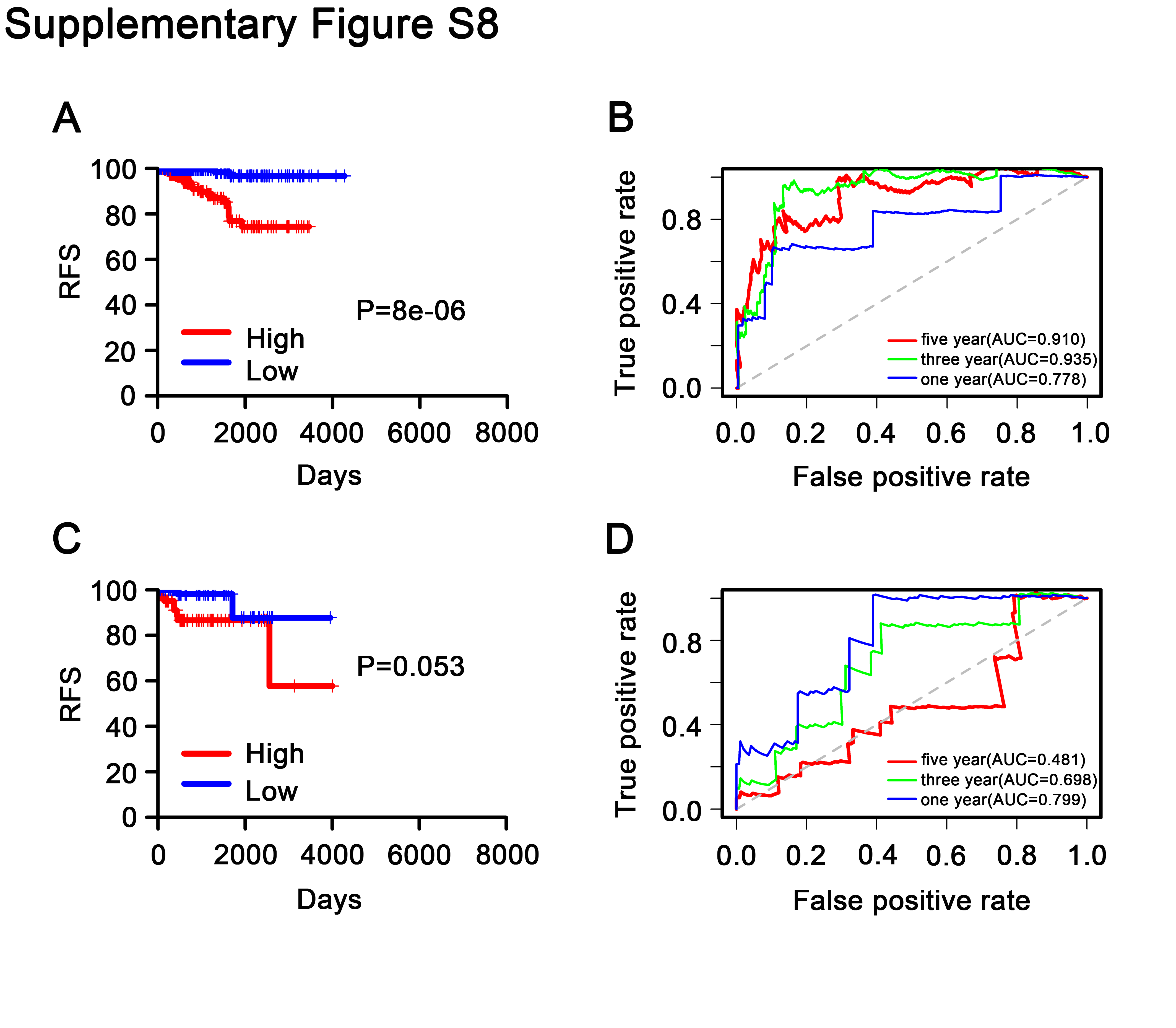

Supplement: Supplementary file 12 [file Image_8.TIF]

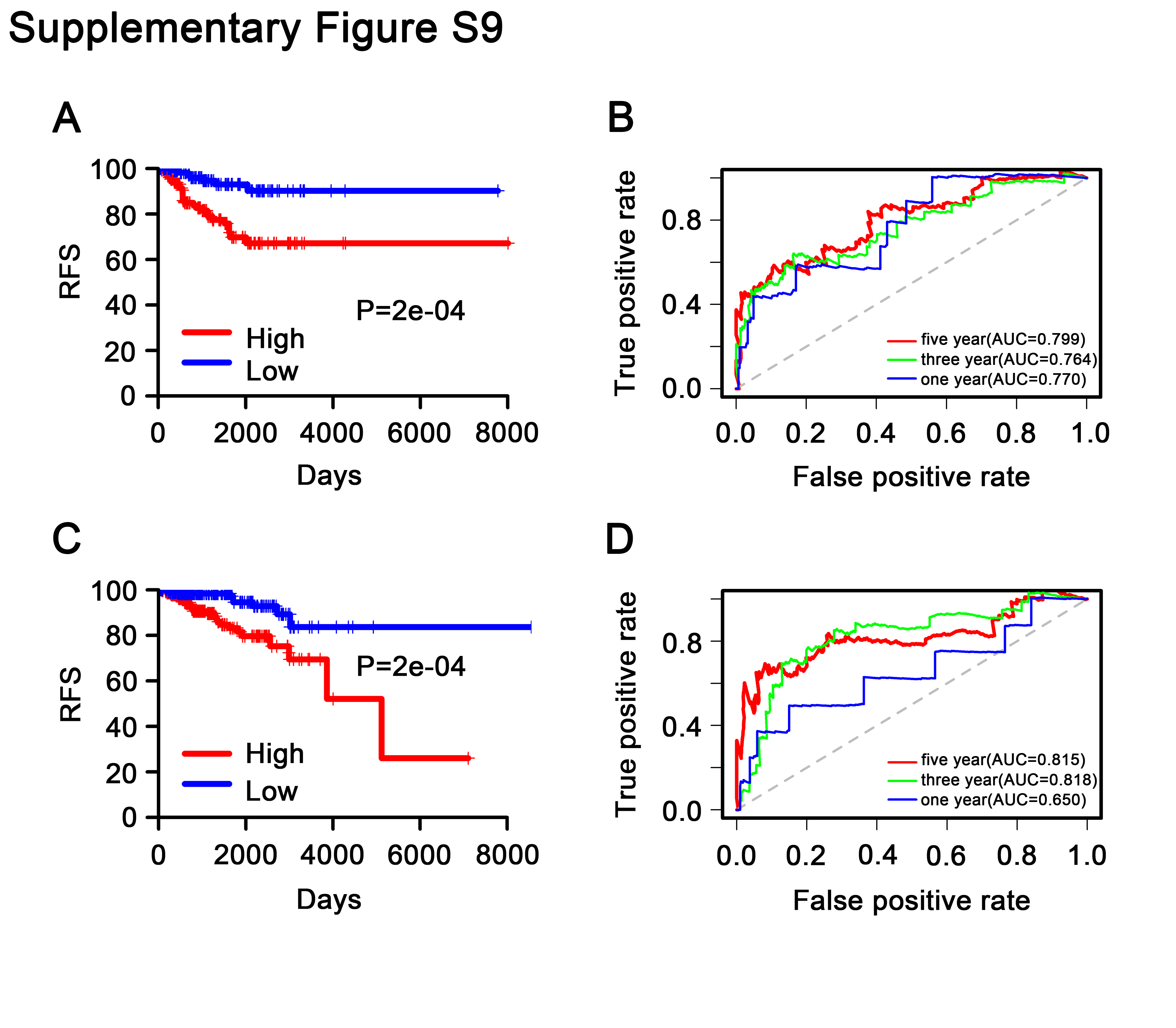

Supplement: Supplementary file 13 [file Image_9.TIF]

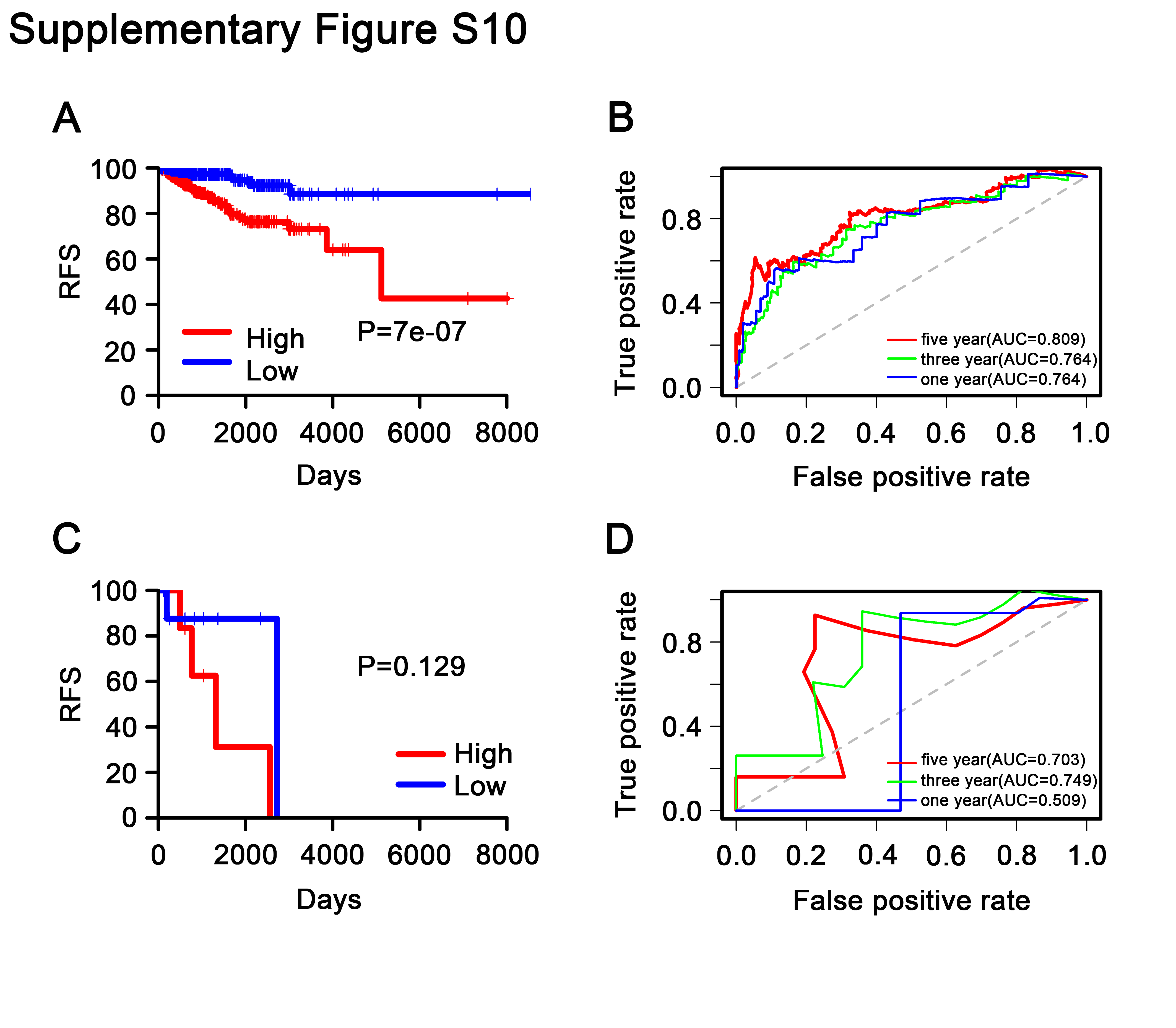

Supplement: Supplementary file 14 [file Image_10.TIF]
